# Vomeric Chondrosarcoma: A Case Report of a Rare Entity With CT and MRI Findings

**DOI:** 10.7759/cureus.99695

**Published:** 2025-12-20

**Authors:** Amine Benfaida, Badr Soudi, El Mehdi Mniai, Samy Ammari, Amal Rami

**Affiliations:** 1 Radiology, Faculty of Medicine, Cheikh Khalifa International University Hospital, Mohammed VI University of Health Sciences, Casablanca, MAR; 2 Otolaryngology - Head and Neck Surgery, Cheikh Zayd University Hospital, Rabat, MAR; 3 Radiology, Mohammed VI Center for Research and Innovation, Cheikh Khalifa International University Hospital, Mohammed VI University of Health Sciences, Casablanca, MAR; 4 Radiodiagnosis, Institut Gustave Roussy, Paris, FRA

**Keywords:** chondrosarcoma, computed tomography, differential diagnosis, magnetic resonance imaging, midfacial degloving, nasal septum, sinonasal tumor, vomer

## Abstract

Chondrosarcoma of the vomer is an exceptionally rare malignant tumor of cartilaginous origin within the sinonasal region. Due to its rarity, the diagnosis can often be challenging, and imaging features may overlap with other cartilaginous or bone lesions. We describe a case of a patient presenting with progressive right-sided epiphora, in whom radiologic and histologic correlation confirmed the diagnosis of vomeric chondrosarcoma. This report highlights the complementary role of CT and MRI in defining tumor extent and guiding surgical management.

## Introduction

Chondrosarcomas are malignant mesenchymal tumors characterized by cartilaginous formation. They comprise 10-20% of primary malignant bone tumors, while constituting only about 0.1% of all head and neck cancers [1]. Within the sinonasal tract, they account for less than 10% of head and neck cases, most frequently arising from the septum or ethmoid [2,3]. Vomeric origin is exceedingly rare, with fewer than 20 cases reported worldwide [4]. This rarity often leads to uncertainty regarding clinical presentation, imaging findings, and therapeutic management. This case report illustrates the diagnostic contribution of radiology and histology in confirming vomeric chondrosarcoma and guiding surgical strategy.

## Case presentation

The patient was a 64-year-old retired female with a medical history notable for thyroiditis, hiatal hernia, peptic ulcer disease, and being overweight, and she had no known allergies. She was a former smoker (20 pack-years, quit at the age of 56 years) and reported no alcohol use. She presented with progressive right-sided epiphora evolving over seven months, which had led her to consult an ENT specialist. Endoscopic examination revealed a lobulated mass arising from the anterior-inferior nasal septum, extending to the nasal floor and displacing adjacent structures without orbital or skull base invasion. Ophthalmologic evaluation was normal, and cranial nerve function remained intact.

**Figure 1:**
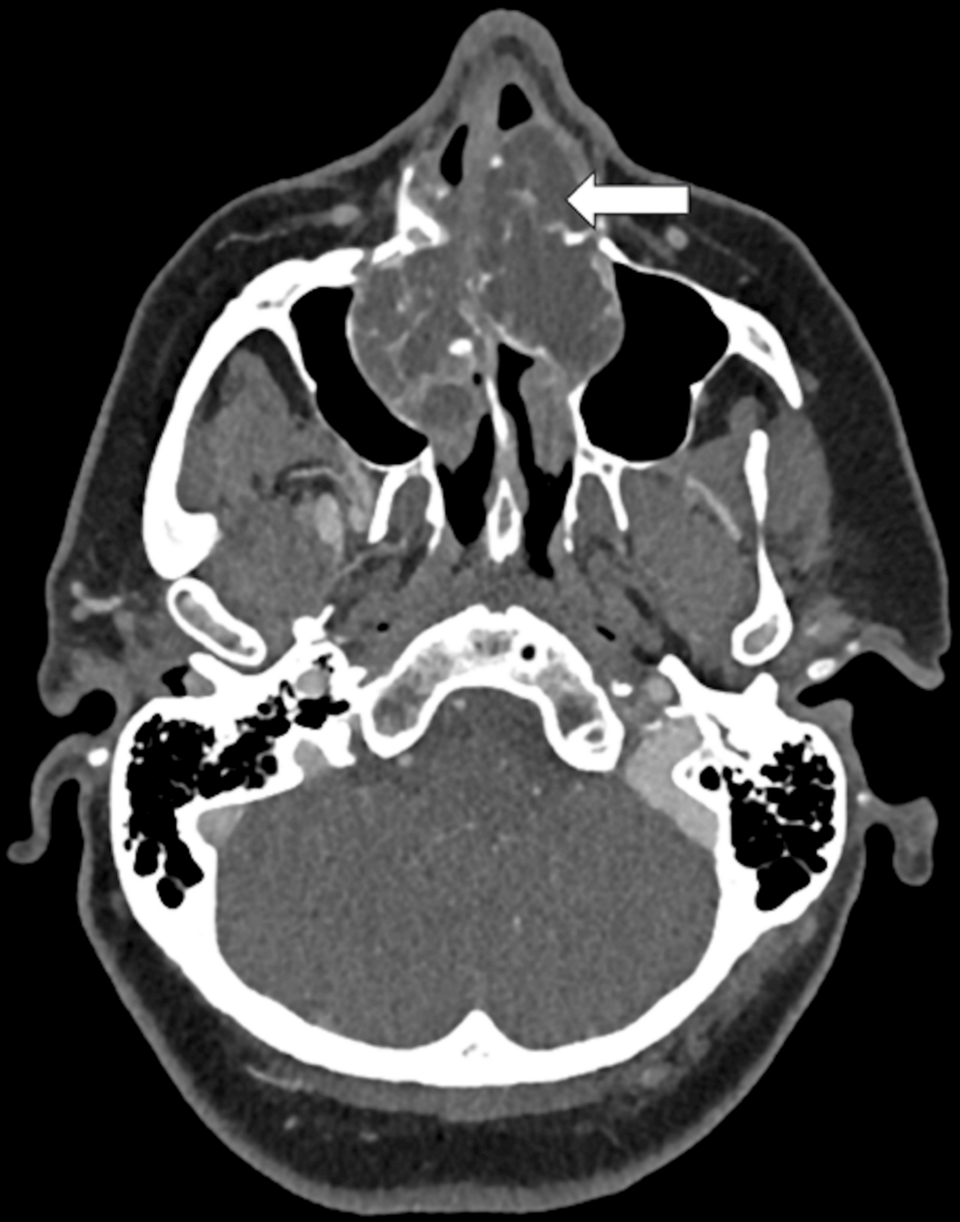
Axial contrast-enhanced CT of sinonasal chondrosarcoma The image shows a large lobulated soft-tissue mass centered on the anterior-inferior nasal septum, extending into both maxillary sinuses and the nasal floor (white arrow). The lesion exhibits internal arciform calcifications typical of a chondroid matrix and causes remodeling of adjacent bony structures CT: computed tomography

**Figure 2:**
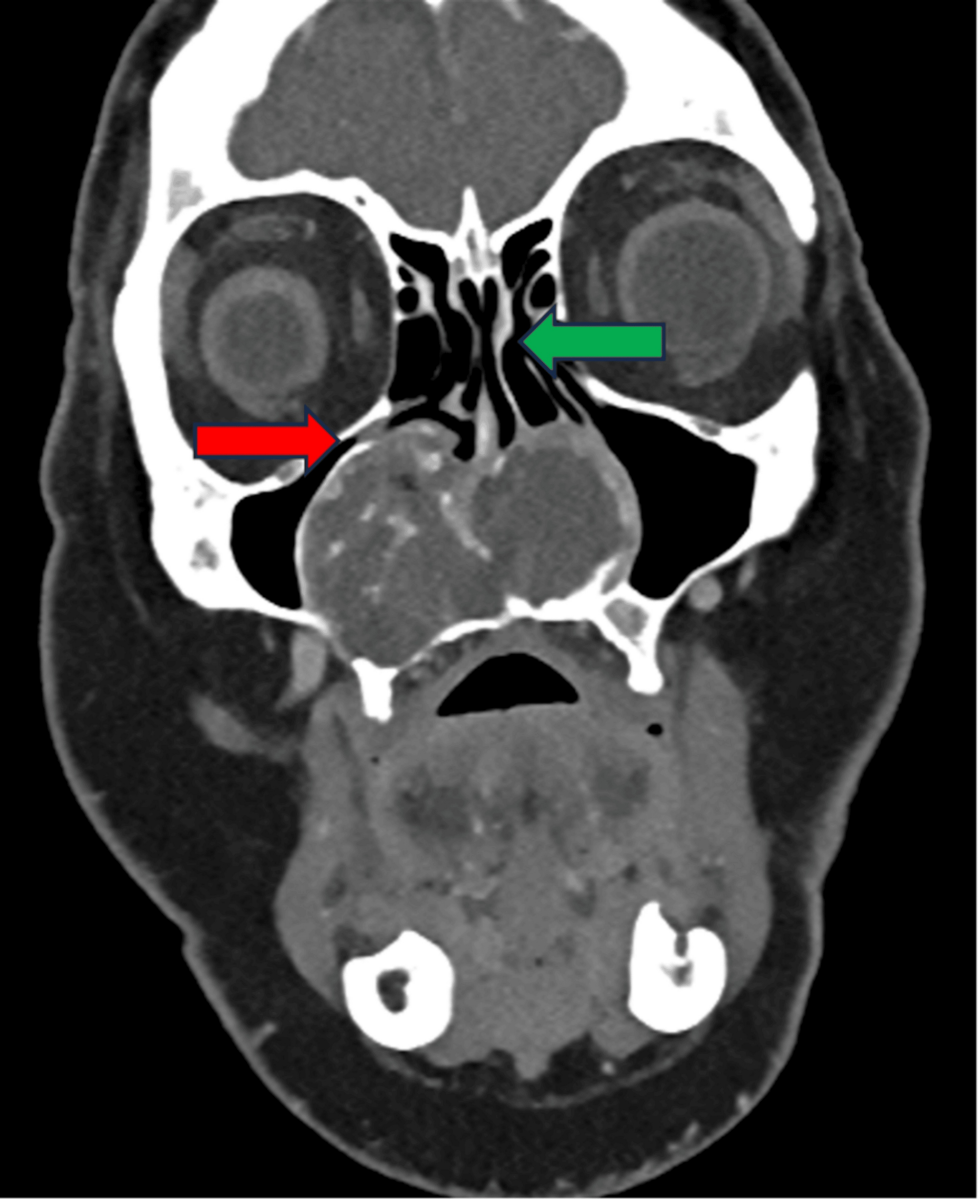
Coronal contrast-enhanced CT of sinonasal chondrosarcoma The image shows a lobulated mass centered on the anterior-inferior nasal septum, expanding into both maxillary sinuses. The lesion spares the orbital floor (red arrow) and the ethmoidal labyrinth (green arrow), confirming the absence of orbital or ethmoidal invasion CT: computed tomography

Contrast-enhanced CT demonstrated a 57 × 48 × 45 mm irregular, polylobulated soft-tissue mass centered on the anterior-inferior aspect of the nasal septum. The lesion contained arciform calcifications consistent with a chondroid matrix and caused remodeling of the surrounding bony structures, expanding into the palate and both maxillary sinuses (Figure 1). Coronal reconstructions clearly showed that the bony walls of both orbits remained intact, confirming the absence of orbital invasion despite the proximity of the lesion (Figure 2).

**Figure 3:**
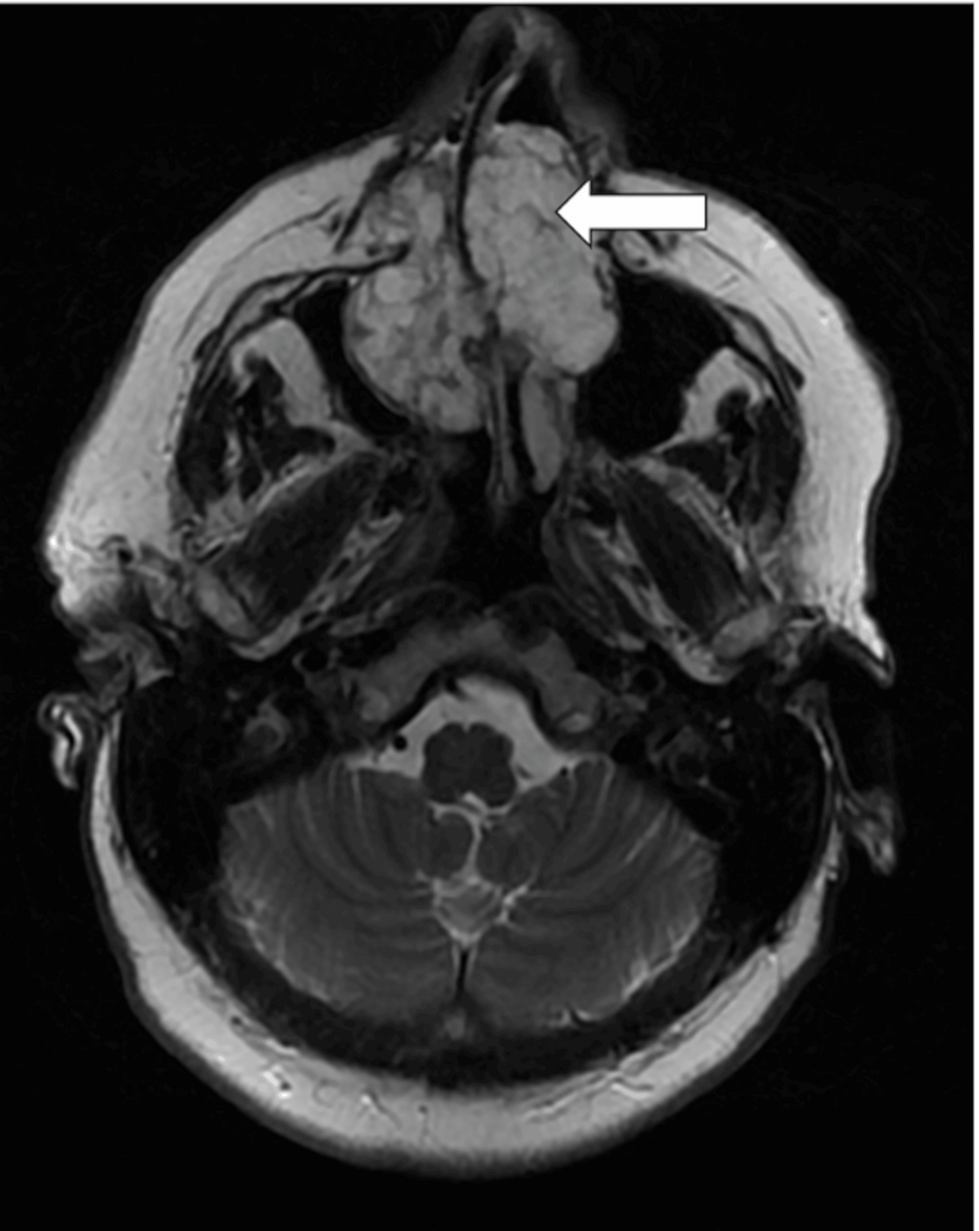
Axial T2-weighted MRI of sinonasal chondrosarcoma The image shows a lobulated mass centered on the anterior-inferior nasal septum, exhibiting marked hyperintensity with internal septations and focal areas of intermediate signal suggestive of chondroid matrix (white arrow). The lesion expands into both maxillary sinuses and the nasal floor, causing remodeling of adjacent structures while preserving the skull base MRI: magnetic resonance imaging

**Figure 4:**
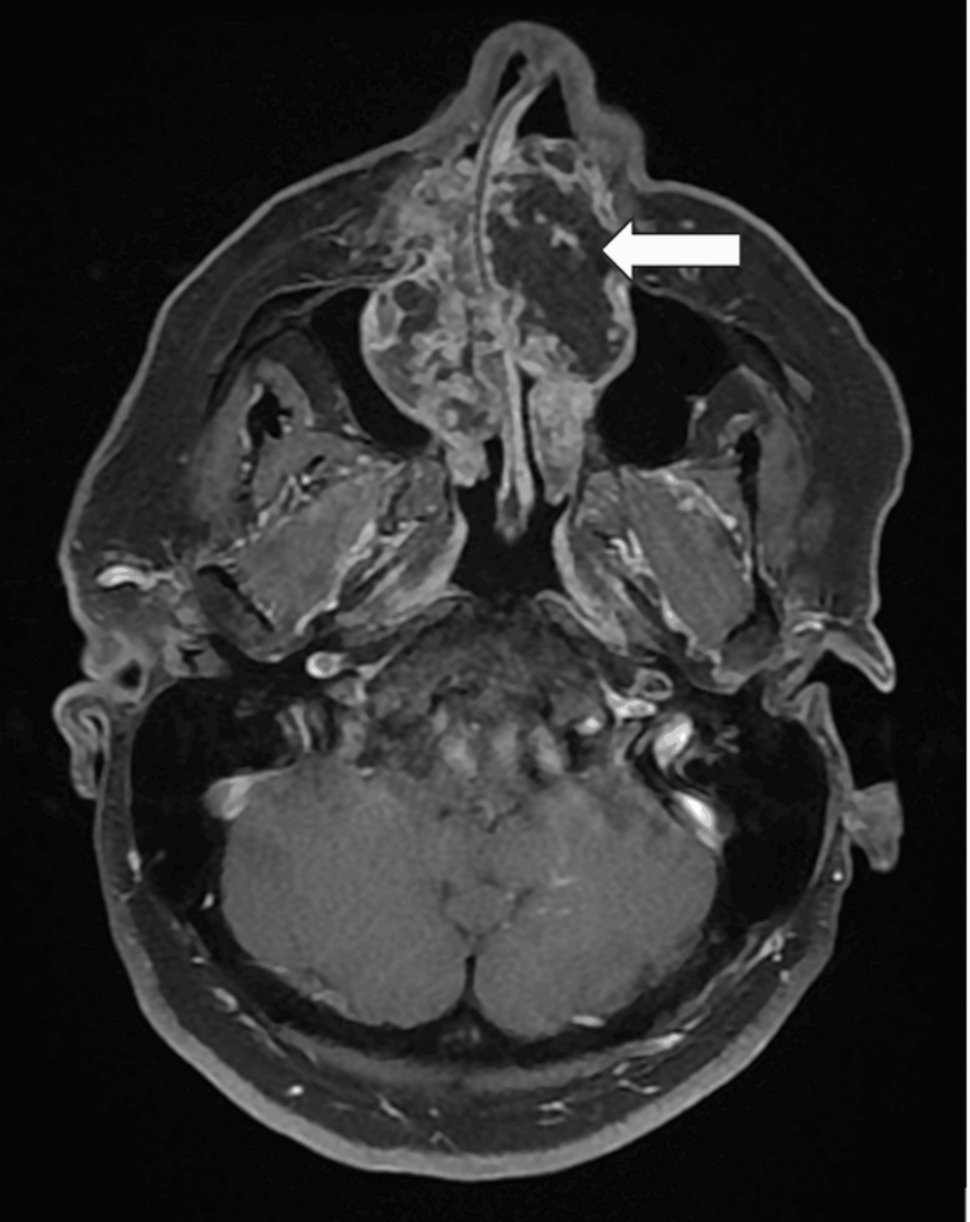
Axial post-contrast T1-weighted fat-suppressed MRI The image demonstrates postoperative changes after subtotal maxillectomy (red arrow) with restoration of the sinonasal anatomy and preservation of the orbital floor. No residual or recurrent enhancing lesion is seen MRI: magnetic resonance imaging

MRI provided better delineation of tumor margins and soft-tissue extension. The lesion appeared markedly hyperintense on axial T2-weighted images with lobulated contours and internal septations (Figure 3). Post-contrast T1-weighted fat-suppressed images showed heterogeneous enhancement with a non-enhancing central component suggestive of cartilaginous or myxoid stroma (Figure 4). The mass filled both maxillary sinuses and inferior turbinates while sparing the ethmoid, orbits, and skull base. Both nasolacrimal ducts were obstructed.

**Figure 5:**
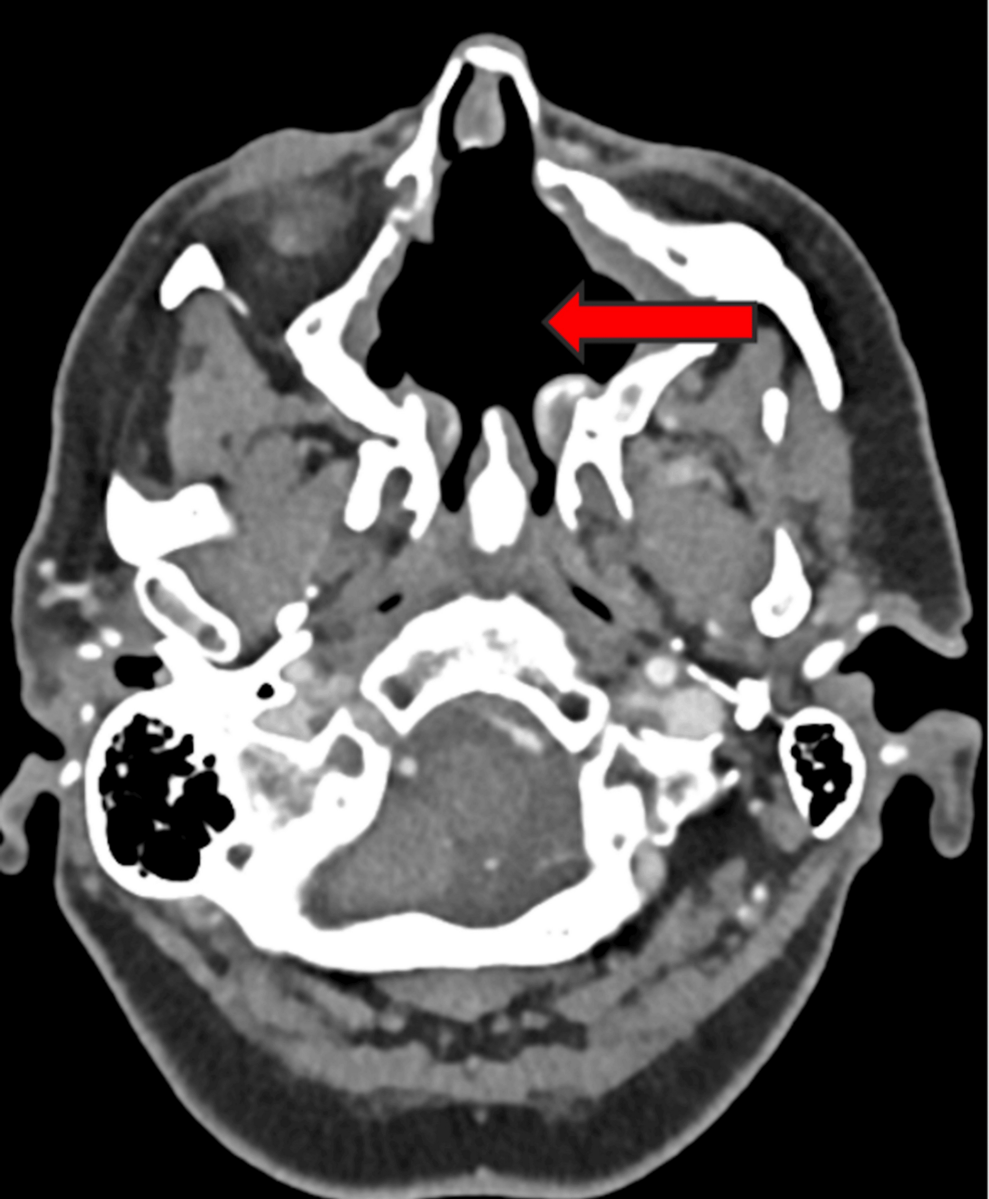
Postoperative contrast-enhanced CT scan of the paranasal sinuses The image demonstrates postoperative changes after subtotal maxillectomy (red arrow) with restoration of the sinonasal anatomy and preservation of the orbital floor. No residual or recurrent enhancing lesion is seen CT: computed tomography

**Figure 6:**
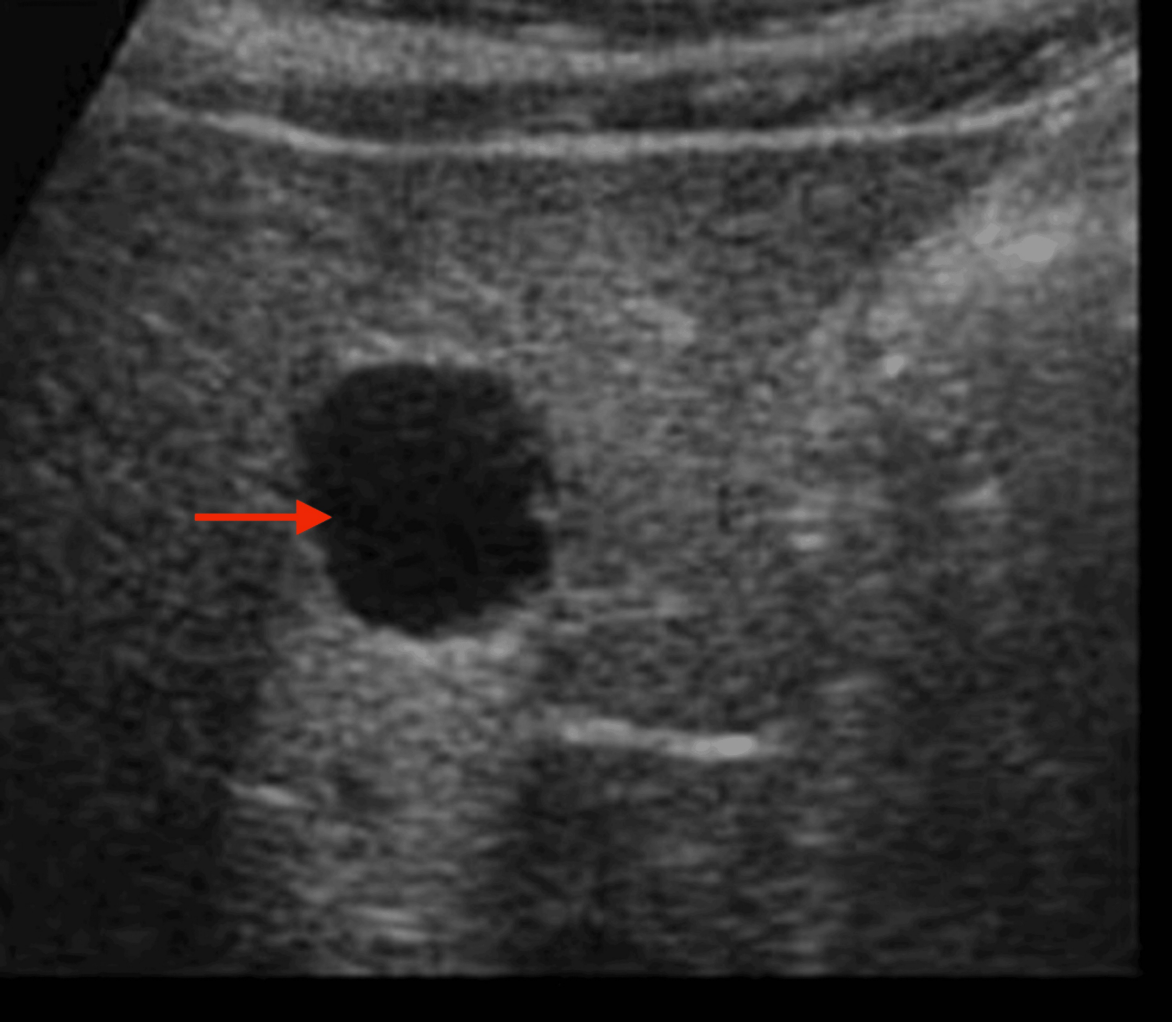
Ultrasound of the liver Transverse abdominal ultrasound showing a well-circumscribed anechoic cystic lesion within the hepatic parenchyma, consistent with a benign simple hepatic cyst (red arrow)

Histopathologic examination revealed a moderately cellular cartilaginous matrix with nuclear atypia and abundant myxoid stroma, consistent with grade II chondrosarcoma. The patient underwent subtotal maxillectomy through a midfacial degloving approach, with preservation of both orbital floors (Figure 5). Reconstruction employed bilateral zygomatic implants and a fascio-cutaneous antebrachial flap to restore nasopalatal continuity. Postoperative recovery was uneventful. Staging excluded distant disease, and a hepatic cyst identified on ultrasound was benign (Figure 6). At the 18-month follow-up, imaging confirmed no evidence of local recurrence.

Unfortunately, the original histopathological slides, intraoperative photographs, and postoperative endoscopic images could not be obtained from the patient’s previous institution, representing a limitation of this case report.

## Discussion

Chondrosarcomas of the sinonasal tract are rare, with an incidence of fewer than 1 per 200,000 cases [1,2]. The nasal septum and ethmoid are the most common sites of origin, whereas involvement of the vomer is exceedingly uncommon [1,3,4]. Most cases arise in the fifth or sixth decade of life, with a slight male predominance [2]. Our patient, aged 64 years, fit this profile but presented with an unusual symptom - chronic unilateral epiphora. This finding reflected obstruction of the nasolacrimal duct, which is a rare mode of presentation. Clinical manifestations depend on the extent of tumor spread. Nasal obstruction, epistaxis, and facial swelling are the most frequent symptoms [2,4]. When epiphora occurs, it results from compression or invasion of the lacrimal pathway. Persistent unilateral obstruction should therefore prompt suspicion of underlying sinonasal disease [5].

Imaging plays a key role in both diagnosis and surgical planning. CT shows osseous remodeling and the arciform or punctate calcifications typical of cartilaginous tumors [5,6]. MRI delineates margins and soft-tissue extension. High T2 signal and heterogeneous enhancement mirror the cartilaginous and myxoid matrix [5,6]. In our patient, CT and MRI mapped palatal and maxillary invasion while confirming sparing of the orbits and skull base - a decisive factor for the surgical approach. The differential diagnosis includes chondroma, chordoma, and chondromyxoid fibroma. Large size, cortical breach, and a permeative growth pattern support chondrosarcoma over benign lesions [6]. Chordoma usually arises in the midline and expresses brachyury, unlike chondrosarcoma [7,8]. Chondromyxoid fibroma is rare in the sinonasal tract and lacks cytologic atypia or permeative invasion [5]. Correlation between imaging and histology establishes the diagnosis, while immunohistochemistry and molecular testing help in ambiguous cases [7-9].

Surgery remains the only effective treatment. Chondrosarcomas are largely resistant to radiotherapy and chemotherapy [1,10]. Complete excision with negative margins is associated with improved survival. Endoscopic resection suits small localized tumors, while open approaches - craniofacial or midfacial degloving - are required for larger or more infiltrative lesions [10]. Similar cases in elderly patients have been reported [11]. Rare cases with orbital or intracranial extension have also been described [12]. Our patient underwent subtotal maxillectomy with bilateral zygomatic implants and an antebrachial flap, restoring both anatomy and function. Radiotherapy may be considered after incomplete resections or in high-grade tumors, while chemotherapy is generally limited to mesenchymal variants [10].

Prognosis depends on histological grade and surgical margins. Reported series indicate five-year survival rates ranging from 50 to 80%, with recurrence rates of up to 40% [1,2]. Ongoing follow-up with endoscopy and imaging is therefore essential. This case reinforces the value of multidisciplinary collaboration between radiologists, surgeons, and pathologists in managing these rare sinonasal tumors. Our findings are consistent with previous reports describing successful surgical outcomes with open or midfacial approaches [11-13]. A major limitation of our case was the unavailability of the original histopathological slides for digital reproduction, although the diagnosis was confirmed by the pathology department.

## Conclusions

Chondrosarcoma of the vomer remains an exceptionally rare tumor of the sinonasal tract. Nonspecific symptoms can complicate the diagnosis. CT and MRI demonstrate its cartilaginous characteristics and delineate tumor extent. Histopathology confirms the diagnosis. Complete surgical resection offers the best outcome, while reconstruction restores both anatomy and function. Long-term follow-up with imaging and endoscopy is essential for early detection of recurrence.
